# Image-Guided Multisession Radiosurgery of Skull Base Meningiomas

**DOI:** 10.3390/cancers12123569

**Published:** 2020-11-29

**Authors:** Alfredo Conti, Antonio Pontoriero, Giuseppe Iatì, Salvatore M. Cardali, Anna Brogna, Filippo Friso, Vittoria Rosetti, Matteo Zoli, Silvana Parisi, Alberto Cacciola, Sara Lillo, Stefano Pergolizzi, Diego Mazzatenta

**Affiliations:** 1 Unit of Neurosurgery, IRCCS Istituto delle Scienze Neurologiche di Bologna, 40139 Bologna, Italy; matteo.zoli4@unibo.it (M.Z.); diego.mazzatenta@unibo.it (D.M.); 2 Dipartimento di Scienze Biomediche e Neuromotorie (DIBINEM), Alma Mater Studiorum University of Bologna, 40126 Bologna, Italy; filippo.friso@studio.unibo.it (F.F.); vittoria.rosetti@studio.unibo.it (V.R.); 3 Department of Radiation Oncology, BIOMORF, University of Messina, via Consolare Valeria 1, 98122 Messina, Italy; apontoriero@unime.it (A.P.); giuseppe.iati@polime.it (G.I.); anna.brogna@polime.it (A.B.); sparisi@unime.it (S.P.); alberto.cacciola0@gmail.com (A.C.); slillo@unime.it (S.L.); Stefano.pergolizzi@polime.it (S.P.); 4 Department of Neurosurgery, BIOMORF, University of Messina, via Consolare Valeria 1, 98122 Messina, Italy; salvatore.cardali@unime.it

**Keywords:** meningioma, skull base, radiosurgery, image-guided radiotherapy, hypofractionated stereotactic radiotherapy, CyberKnife

## Abstract

**Simple Summary:**

Stereotactic radiosurgery has changed the landscape of treatment for skull base meningiomas. Lesions encasing or compressing radiosensitive structures are considered not suitable for single session stereotactic radiosurgery because of the high risk of side effects. Multisession stereotactic radiosurgery can reduce these risks, allowing for normal tissue repair between fractions, while delivering a high dose per fraction. The aim of this study is to validate the role of multi-session stereotactic radiosurgery in the treatment of skull base meningiomas, through a retrospective analysis of 156 patients affected by skull base meningioma, treated at the University of Messina between 2008 and 2018. Our study suggests that multisession stereotactic radiosurgery represents a safe and effective profile in the treatment of skull base meningioma, providing a satisfactory local control and a low toxicity rate, together with patient comfort from a frameless procedure.

**Abstract:**

Background: The efficacy of single-session stereotactic radiosurgery (sSRS) for the treatment of intracranial meningioma is widely recognized. However, sSRS is not always feasible in cases of large tumors and those lying close to critically radiation-sensitive structures. When surgery is not recommended, multi-session stereotactic radiosurgery (mSRS) can be applied. Even so, the efficacy and best treatment schedule of mSRS are not yet established. The aim of this study is to validate the role of mSRS in the treatment of skull base meningiomas. Methods: A retrospective analysis of patients with skull base meningiomas treated with mSRS (two to five fractions) at the University of Messina, Italy, from 2008 to 2018, was conducted. Results: 156 patients met the inclusion criteria. The median follow-up period was 36.2 ± 29.3 months. Progression-free survival at 2-, 5-, and 10- years was 95%, 90%, and 80.8%, respectively. There were no new visual or motor deficits, nor cranial nerves impairments, excluding trigeminal neuralgia, which was reported by 5.7% of patients. One patient reported carotid occlusion and one developed brain edema. Conclusion: Multisession radiosurgery is an effective approach for skull base meningiomas. The long-term control is comparable to that obtained with conventionally-fractionated radiotherapy, while the toxicity rate is very limited.

## 1. Introduction

### 1.1. Background/Rationale

Skull base meningiomas represent one of the greatest neurosurgical challenges, especially when they encase multiple neurovascular structures. The involvement of this critical structures makes complete surgical resection occasionally impossible or associated with a high risk of neurological sequelae. The introduction of stereotactic radiosurgery (SRS) has changed the landscape of treatment for such lesions. Multiple retrospective studies have shown the safety and efficacy of the SRS approach for meningiomas, highlighting its role in the upfront treatment of selected cases and also as an adjuvant treatment for residual or recurrent tumors. The tumor control rates for WHO Grade-I skull base meningiomas after SRS are approximately 91% and 88% at 5 and 10 years, respectively [1,2,3,4,5,6,7,8,9,10,11].

Single-session SRS (sSRS) has been limited to the treatment of small- to moderately-sized lesions. Traditionally, a tumor diameter of 30–35 mm was recommended as the cut-off for radiosurgery [5,11,12]. Furthermore, lesions encasing or compressing radiosensitive structures, like the optic nerves, optic chiasm, and the brainstem, are not considered suitable for sSRS.

The emergence of frameless image-guidance technology enabled the principles of multisession stereotactic treatments, conventionally consisting of two to five fractions of 4–10 Gy each. The goal of this technology is the delivery of a highly conformal irradiation, actually comparable to that of sSRS, with an assumed lower toxicity on the critical neural structures [13,14,15]. Multisession stereotactic radiosurgery (mSRS) can reduce the risk of late side effects, allowing normal tissue to repair between fractions, while delivering a high dose per fraction. In addition, reoxygenation and reassortment between fractions can improve tumor control by increasing the cell kill rate. Recently, short- to mid-term follow up data on mSRS of meningiomas have been reported [13,16,17,18,19,20,21,22,23,24,25]. The results are encouraging, but several issues remain to be definitively addressed. In particular, the radiobiological bases and the dose/fraction schemes to be used in the hypofractionated treatment of meningiomas still need to be clarified [13,26]. Moreover, only small series with limited follow up have demonstrated the overall safety and efficacy of mSRS.

### 1.2. Objective

The aim of this study was to evaluate the role of mSRS in the treatment of skull base meningiomas, through a retrospective analysis of a large series of patients treated with multisession frameless robotic radiosurgery (CyberKnife, Accuray Inc., Sunnyvale, CA, USA). The results are reported at a moderately long term.

## 2. Material and Methods

### 2.1. Study Design

We retrospectively reviewed the clinical, radiological, and surgical outcomes of patients with skull base meningiomas treated by mSRS (two to five fractions) using a CyberKnife system, between January 2008 and December 2018.

### 2.2. Setting

Patients were recruited and treated at the CyberKnife Center of the University of Messina, Messina, Italy.

### 2.3. Inclusion Criteria

Inclusion criteria: (I) histologically verified, or clinically and radiologically supposed diagnosis of WHO I meningioma (no evidence of tumor growth, >10% of the pre-treatment volume, in two sequential MR controls six months apart), (II) age >18 years, (III) meningioma clearly originating from the base of the skull, (IV) irradiation schedule in multiple fractions, and (V) availability of complete pre- and post-operative clinical and radiological data.

### 2.4. Participants and Sample Size

More than 500 patients affected by meningioma were treated. Among these, a cohort of 156 patients affected by skull base meningiomas treated with mSRS were included in this analysis. The clinical and demographic characteristics, as well as the treatment parameters, are summarized in Table 1.

### 2.5. Ethics

This study was approved by the Local Ethics Committee (Comitato Etico Interaziendale della Provincia di Messina; http://www.polime.it/comitato_etico_interaziendale) Prot. 0012782.

### 2.6. Imaging and Treatment

An image-guided, frameless 6 MV radiosurgery system, namely a Cyberknife SRS system, was used for the treatment; in brief:

#### 2.6.1. Neuroimaging

The neuroimaging technique consisted of a thin-section, contrast-enhanced, multiplanar reconstruction-gradient echo volumetric MR study performed with the following parameters: TR 9.7 ms, TE 4 ms, matrix 200 × 256, flip angle 1, orientation sagittal. The CT protocol was elaborated according to the CyberKnife-specific requirements: acquisition 16 × 0.75 mm, Kv 120, mAs 320, rotation time 1 s, pitch 0.45; and reconstruction slice 1 mm, reconstruction increment 1 mm, filter reconstruction B30 (smooth), 512 × 512 matrix. The axial source images were transferred to the CyberKnife workstation. 

#### 2.6.2. The Planning Procedure Included Several Sequential Steps

**Figure 1:**
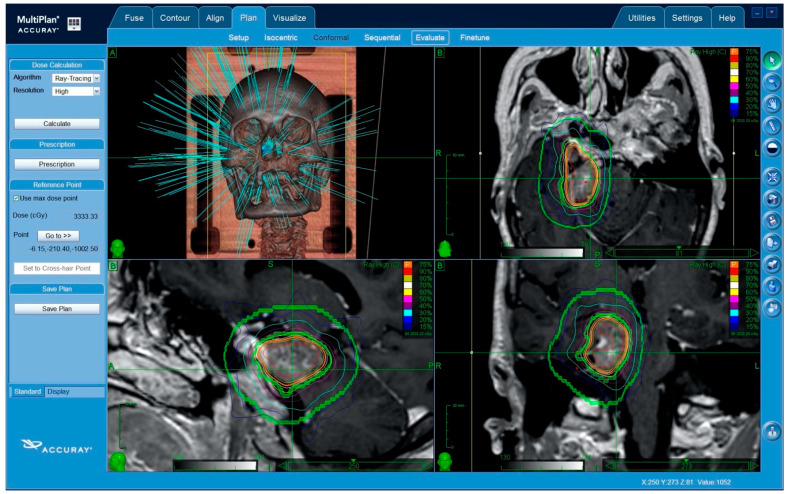
Treatment plan for a petroclival meningioma, showing some of the main steps. Single beams of radiation penetrate mainly through the splanchnocranium so as to protect the brain. Trajectories through the eyeballs are excluded. The use of tuning structures (green circles): these tuning shells are used as artificial critical organs in the inverse planning algorithm. A specific dose is set for each shell in order to contain the lower dose distribution outside the planning treatment volume (PTV). This is a fundamental step of non-isocentric planning, in order to force the system to use a wide array of beams (hundreds) and to avoid dangerous peripheral radiation hot-spots.

(1) *Contouring*: the contouring of the tumor and the critical volumes was performed on co-registered MR-CT images. The volumes were manually outlined on axial images, with a simultaneous overlay of these contours on coronal and sagittal reconstructions. In all of the cases, the planning treatment volume (PTV) and gross tumor volume (GTV) coincided.
(2) *Dose selection*: the marginal and maximal dose, as well as the number of sessions were influenced by a multitude of factors, including patient age, tumor volume, length and volume of the irradiated critical organs (optic nerve, chiasm, or brainstem), and neurological function. Adopting an α/β of 2, the target biologically equivalent dose (BED) to be used in the different fractionation schedules was 87.5 Gy_2,_ corresponding to that of a 25 Gy in a five fractions schedule [13,27]. The dose to any portion of the anterior visual pathway was set not to exceed a maximum of 500 cGy per fraction [13].
(3) *Planning*: An inverse planning algorithm using a nonisocentric technique determined the optimal treatment planning program. The ray-tracing algorithm was routinely used for this purpose. Some of the methods used included the following: (a) selection of the size and number of collimators, balancing the necessity of target coverage, reduction in the number of radiation beams, and monitor units with the necessity of steep dose gradients in specific areas; (b) the addition of tuning structures to reduce uncontrolled dose diffusion (Figure 1); (c) definition of dose constraints and their weight to the target volume and critical structures; and (d) maximization of the resolution of a dose calculation using the smallest calculation grid. (e) Plan evaluation, including: a visual analysis of the dose–volume histograms for the target and all of the critical structures, and evaluation of the PTV coverage and the dose conformity. Conformity was expressed with respect to the PTV as the new conformality index (nCI), defined as the inverse Paddick conformity index [28], where

(1) $$nCI=\frac{V_{P}}{V_{PTV,P}}\frac{V_{PTV}}{V_{PTV,P}}$$

with the PTV volume being *V_PTV_*, the volume covered by the prescription dose *V_P_*, and the volume of PTV covered by the prescription dose *V_PTV,P_*. The coverage was defined as the fraction of the PTV covered by the prescribed dose. The *nCI* was kept as low as possible, whereas coverage was kept as close to 100% as possible; (f) the calculation grid was then expanded to evaluate the distant isodose distribution.

#### 2.6.3. Treatment Delivery

Prior to the treatment delivery, patient alignment to treatment position was performed by registering live stereoscopic X-ray images to the digitally reconstructed radiographs (DDRs) obtained from the pre-treatment CT, using bony anatomy. During the treatment delivery, the intrafraction motion was tracked by comparing the live X-ray images to the corresponding DRRs using dedicated image-guided tracking algorithms. The fractions were delivered 24 h apart.

### 2.7. Data Sources and Patient Assessment

All of the radiometric, clinical, and follow-up data were obtained from the CyberKnife institutional database. All patients underwent serial radiological and neurological evaluations, and, in selected cases, endocrinological and ophthalmological examinations. Radiological assessment consisted of a contrast-enhanced MRI scan obtained at 3 months, then every 6 months for 2 years, and then yearly. At follow-up, contrast enhanced T1 weighed, T2w, Proton Density, and fluid-attenuated inversion recovery (FLAIR) sequences were performed in all cases.

Ophthalmological follow-up was obtained in peri-optic tumors, i.e., tumors lying within 2 mm of the optic apparatus. Visual acuity studies and a computerized visual field perimetry test were periodically performed by an ophthalmologist. Visual acuity was considered normal if >20/80; the visual perimetry was qualitatively assessed. The first assessment was obtained 6 months after the treatment, then every 6 months for 2 years and then yearly. Thyroid hormones, prolactin, cortisol, dehydroepiandrosterone (DHEA), and IGF-1 serum levels were monitored at the same time intervals as the ophthalmological follow-up. Normal range values could differ depending on laboratories and were, therefore, evaluated case by case.

### 2.8. Bias and Assessment of Outcome Variables

In order to avoid inconsistent interpretation, the clinical results were evaluated according to the numerical values and scales, when applicable. The variables that were examined were the (I) size of the lesion, (II) occurrence of neurological deficits, (III) presence of radiation-induced complications, and (IV) Karnofsky Performance Status (KPS).

The radiological and clinical follow-up data were collected and stored during outpatient visits. A multidisciplinary tumor board reviewed and discussed these data. In particular, the final evaluation of disease stability or progression was provided by a team of neuro-radiologists. Tumor progression was defined as any increase in size of the tumor detected upon pre-treatment imaging. The treatment was aimed at the stabilization or shrinkage of the tumor, including any contrast enhancing area surrounding the main tumor bulk. Treatment failures were defined as a consistent increase in the size of the treated tumor (>10% of the pre-treatment volume), persisting or further progressing in two sequential MR control, 6 months apart. Marginal progression (i.e., within 10 mm of the target volume) were also considered to be treatment failure.

The progression-free survival (PFS) was calculated according to the Kaplan–Meier method, and was expressed as the time between initial treatment and the demonstration of any tumor progression after mSRS. Every in-field or marginal progression was calculated in the Kaplan–Meier curve analysis. 

### 2.9. Statistical Methods

The patients’ characteristics were presented as percentages for the dichotomous data, and as means with standard deviations or medians with value ranges for continuous data. Progression-free survival (PFS) with the corresponding survival curves and probabilities at different time points were estimated using the Kaplan–Meier method. Curve comparisons were performed by log-rank (Mantel-Cox) test. Data were analyzed using SAS software (SAS^®^ 9.3, SAS Institute, Cary, NC, USA).

## 3. Results

### 3.1. Participants

More than 500 patients with a diagnosis of meningioma underwent radiosurgical treatment during the considered period. Of these, 156 patients met the inclusion criteria. There were 37 men and 119 women. The mean age at presentation was 58 ± 11.4 years, (median 58 years, range 30–81 years). Before treatment, a diagnosis of benign meningioma (WHO grade I) was histologically obtained in 49 patients and supposed in 107 patients. This was based on the volumetric stability in the sequential MR studies. No patient included in this series showed a rapid radiological progression (i.e., >10% of the pre-treatment volume in two sequential MR control 6 months apart) excluding 2 patients who presented a rapid and uncontrolled progression and underwent rescue surgical resection. The histological analysis demonstrated a WHO grade II meningioma. These two patients were, therefore, excluded from this study. The mean follow-up period was 36.2 ± 29.3 months (median 36.6 months; range 2–137 months). Before treatment, 35 patients (22.4%) complained about cranial neuropathy (Table 1).

### 3.2. Descriptive Data of Radiosurgical Doses and Schedules

**Figure 2:**
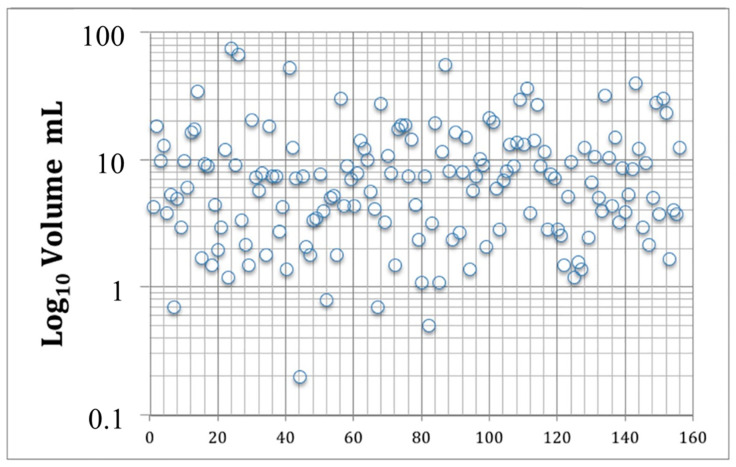
Final planning treatment volumes (PTVs) of all patients in the series. On the X axis, numbers indicate individual patients.

In this series, all of the patients received mSRS. Treatment and dosimetric characteristics are summarized in Table 2. The average gross tumor volume (GTV) was 10.3 ± 11.9 mL, with a median of 7.5. The GTV and the planning treatment volume (PTV) corresponded in all of the cases (Figure 2).

**Figure 3:**
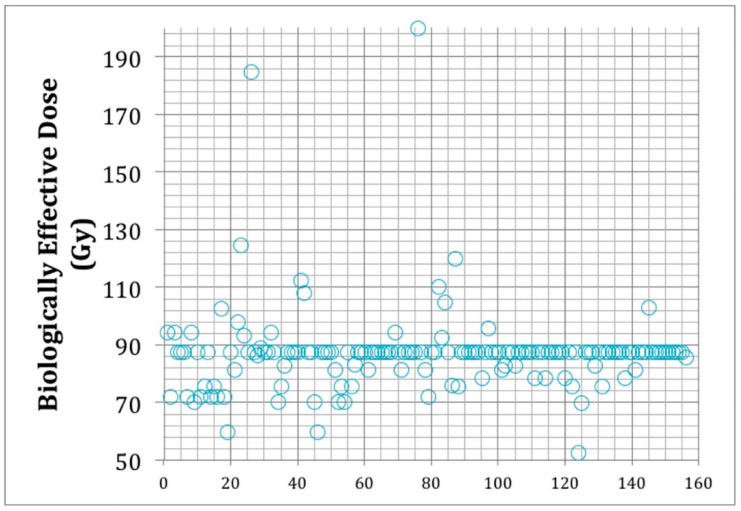
Biologically effective doses (BED) adopted in individual patients (each represented by a circle) in this series. The BED was calculated using the linear/quadratic (LQ) model and an alfa/beta of 2 for benign meningiomas. The figure shows that most patients were treated with a median BED of 87.5 Gy. On the X axis, numbers indicate individual patients.

Mean dose was 25 ± 5.3 Gy, and the median dose was 25 Gy. The median number of fractions was 5 ± 2. The median biologically effective dose (BED) was 87.5 ± 15.1 Gy (Figure 3). The median equivalent dose to the conventional fractionation (EQD2) was 43.8 ± 7.6 Gy.

The prescription isodose ranged from 62% to 86% (median 77 ± 5.1%). The median number of beams was 190 ± 26.5. The median nCI was 1.4. The median maximum point dose to the PTV was 32.6 Gy (mean 32.1 Gy ± 6.4 Gy). The median maximum point dose to the optic chiasm and optic nerves were 25 Gy and 25 Gy, respectively.

### 3.3. Tumor Control

The local control in the whole series was 95%, with a median follow up of 36.6. Ninety five out of 156 patients (60.9%) showed a stable disease or a minimal response (<10% size decrease); 53/156 patients had a partial response (34%) (<50% size decrease); 8/156 patients (5.1%) suffered from a progressive disease. In 3.8% (6/156 patients), progression was in-field, whereas in the remaining 1.3% who experienced a progressive disease (2/156), the progression was marginal (within 10 mm from the prescription isodose line).

**Figure 4:**
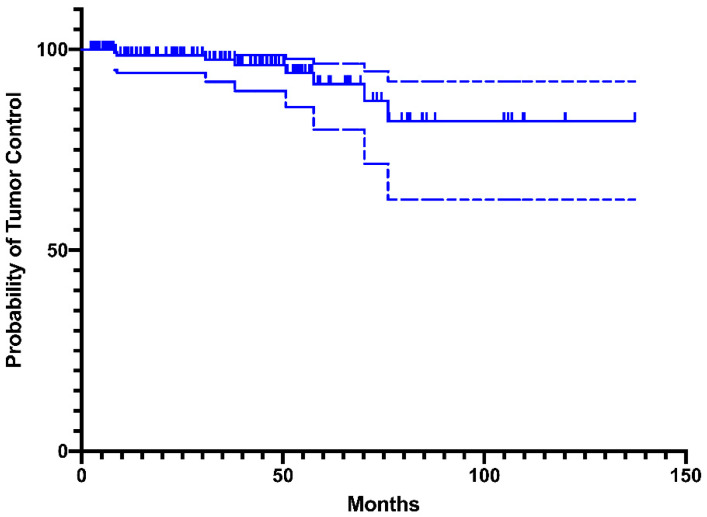
Progression-free survival (PFS) ± 95% confidence interval.

According to the Kaplan–Meier analysis, the progression-free survival at 2, 5, and 10 years was 95%, 90%, and 80.8%, respectively (Figure 4).

### 3.4. Clinical Outcome

According to the pre-treatment evaluation, 35 patients (22.4%) had some cranial nerve deficit; 8.3% suffered a visual field deficit and/or a visual acuity loss, trigeminal neuralgia was described by nine patients (5.8%), diplopia was present in seven patients (4.5%), facial paresis in three patients (1.9%), hearing impairment in two patients (1.3%), and one patient (0.6%) had swallowing difficulties. 

Following mSRS, 15 of the 35 patients (42.8%) with cranial neuropathy at the time of treatment improved at the last clinical follow-up. Nine patients (5.8%) had a new cranial neuropathy. In all of these cases, the neuropathy was transient and included trigeminal neuralgia. The neuralgia was transient, and could be controlled with less than 400 mg of carbamazepine daily, except in one patient affected by a pre-existing trigeminal neuralgia that occurred after previous irradiation. No other permanent cranial neuropathy cases were detected. One patient with a large sphenopetroclival meningioma encasing the carotid artery developed its occlusion and a transient facial nerve deficit. 

Other pre-treatment clinical symptoms were headache (23 patients, 14.7%) and dizziness (10 patients, 6.4%). No patients developed new pituitary deficits.

One patient with a large sphenopetroclival meningioma developed persisting edema in the temporal lobe, causing papilledema that required prolonged steroid administration.

Eight patients (5%) underwent surgical resection as a result of tumor progression.

At the baseline and at last clinical follow-up, the mean KPS score was 88 ± 9.1, with a median of 90 (range 70–100) and 90 ± 9.6 (median 90) points, respectively (*p* = not significant). There was no mortality related to the meningioma during the follow-up period. No other adverse events (>grade 2) were registered in this series. 

### 3.5. Upfront versus Adjuvant Treatment

**Figure 5:**
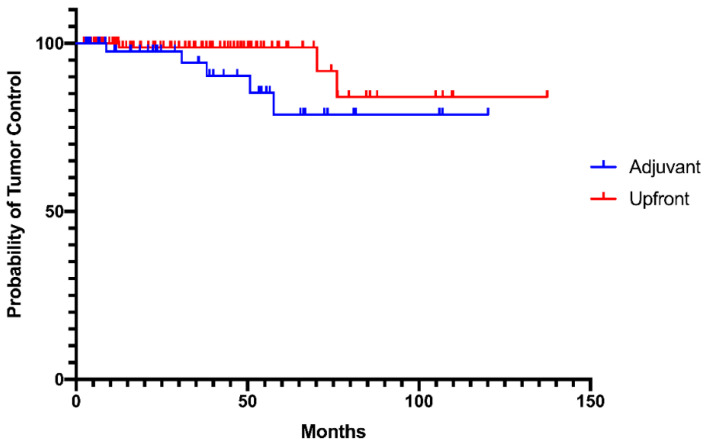
Progression-free survival (PFS) of patients undergoing mSRS as upfront (red) and adjuvant (blue) treatment. Log-rank (Mantel–Cox) test *p* = 0.08.

One-hundred and seven patients underwent upfront radiosurgery, whereas 49 underwent adjuvant treatment. Among these 49 patients, 22 underwent radiosurgery within 6 months after surgery because of a residual tumor; 27 were treated post-surgery, but at the time of radiological recurrence or after progression of post-surgical remnant. When comparing adjuvant versus upfront treatment, the two groups did not differ in terms of median prescription dose, median number of fractions, median BED, and complication rate. There was a significant difference between mean PTV, which was 8.9 cc in the adjuvant group and 13.3 cc in the upfront group (*p* = 0.03). Tumor control was similar in the two groups, although we recorded a tendency toward a lower control rate in the group treated upfront (*p* = 0.08; Figure 5). 

## 4. Discussion

### 4.1. Key Results

This study confirms that multisession SRS is a valid, highly effective, and well-tolerated treatment option for patients with skull base meningiomas, that are neither candidates for surgical resection nor suitable for sSRS. Indeed, image-guided SRS delivered in multiple sessions (median of five fractions) led to a beneficial risk–benefit profile in these patients. In our series, the local control at 2-, 5-, and 10-year follow up was 95%, 90%, and 80.8%, respectively. These results are in line with those reported in previous studies of meningiomas treated using both sSRS for smaller tumors or fractionated radiotherapy for larger tumors.

### 4.2. Interpretation

Radiation therapy is an effective alternative treatment in patients with skull base meningiomas, either as single-fraction radiosurgery or with a conventional fractionation. Local control rates in fractionated regimens range between 80% and 100%, depending on the size of the lesions, location, dose applied, and length of follow-up [29,30,31,32,33,34,35,36,37,38,39,40,41,42,43,44,45]. Modern radiotherapy techniques including intensity-modulated radiotherapy (IMRT) and fractionated stereotactic radiotherapy (FSRT) may offer a favorable treatment profile and outcomes, as compared with the more traditional 3D conformal radiotherapy, through the use of image-guidance and improved conformal dose distributions. Several studies suggest that both FSRT and SRS are valid alternatives to surgical resection in selected skull base meningiomas, providing comparable satisfactory long-term tumor control [8,46,47,48]. Local tumor control rates with FSRT and SRS were reported to be 93–97% and 90–94%, respectively [8,46,47,48]. The permanent morbidity rate was similar, between 0% and 2.6% for both FSRT and SRS. According to the current practice [8], selection between FSRT and SRS depends mostly on the diameter of the meningioma and the distance from radiation-sensitive structures. Indeed, in meningiomas with a diameter of <3 cm and lying more than 3 mm away from radiosensitive structures, SRS is generally preferred. FSRT is typically performed for all tumors not amenable to SRS. 

The use of hypo-fractionated or multisession radiosurgery was introduced in the last years, made possible by advanced image-guidance systems. The Cyberknife (Accuray, Sunnyvale, CA, USA) is a frameless, LINAC-based system that delivers nonisocentric, single beams of radiations through a high number of penetration trajectories from a robotic delivery system [49]. The real-time image guidance, based on the constant acquisition of the skull position by two perpendicular sources of X-rays, provides an accurate localization capability to treat a brain target without the need for a rigid frame with submillimetric accuracy and highly conformal dosimetry. This real-time guidance allows for an accurate patient set-up between fractions and for the detection of intrafraction motion for frameless irradiation [49].

**Figure 6:**
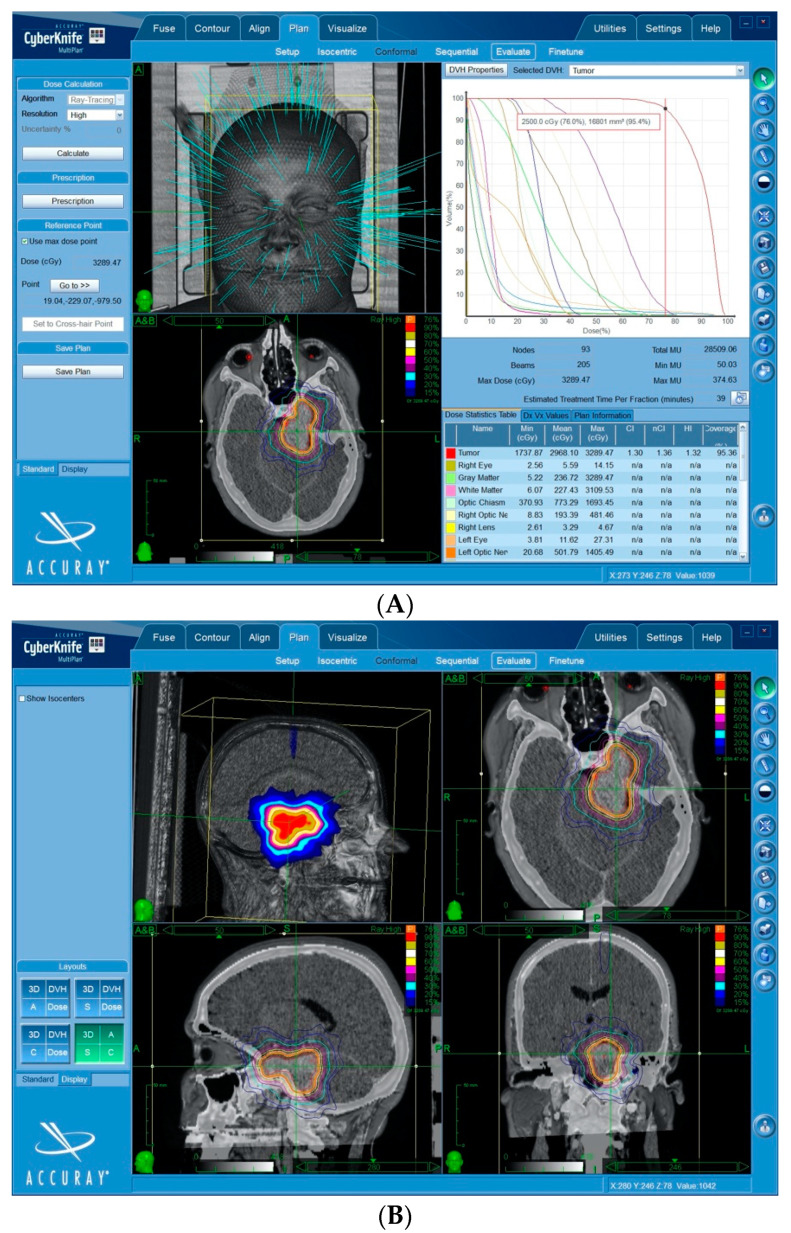

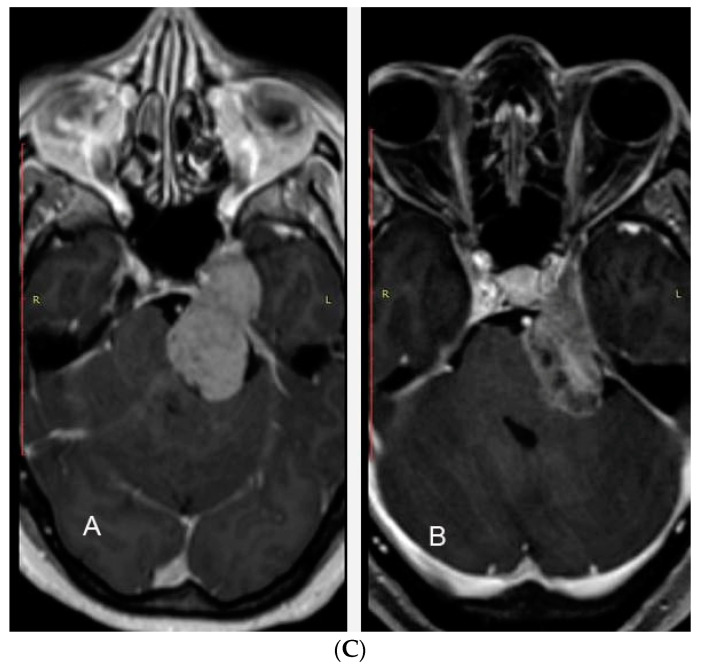
A large petroclival meningioma treated by mSRS. (**A**) The dose–volume histograms quantify the treatment characteristics. The prescription dose was 25 Gy in five fractions. The PTV was 16.8 cc. The nCI was 1.3 and the tumor coverage 95.4%. Maximal doses to the optic chiasm and left optic nerve were 16.9 Gy and 14 Gy, respectively. (**B**) Doses distribution in the three spatial planes showing the rapid dose fall-off from 80% to 15%, despite the large volume of the lesion. (**C**) Pre-treatment MR (**A**) and 3-year control MR (**B**) showing satisfactory tumor shrinkage.

Using multiple fractions, high doses can be applied to virtually all sized lesions and to lesions close to the anterior optic pathway, representing an alternative to conventionally fractionated radiotherapy (i.e., 25 to 30 fractions of 1.8–2.0 Gy; Figure 6).

Beyond specific CyberKnife technology, multisession or hypofractionated SRS have also been performed with the same platforms used for sSRS [15,16,25,50,51], demonstrating similar local control rates compared to single fraction treatment, and may present a lower risk of side effects. Unger and colleagues [51] analyzed 173 patients with meningiomas, and reported that the 56% of patients underwent single fraction radiosurgery with a Gamma Knife and the remainder percentage received multisession radiation therapy over generally two to five fractions with a CyberKnife. The median dose for SRS was 15 Gy and the usual regimen for mSRS was 25 Gy in five fractions. Two-year actuarial risk of symptomatic edema was 3.2% for multisession stereotactic radiation therapy, and 12.5% for SRS. A tumor size greater than 4.9 cm^3^ was also a significant predictor of symptomatic edema [51]. Multisession SRS has typically been adopted for the treatment of peri-optic meningiomas. Midterm follow-up data on multisession radiosurgery of peri-optic meningiomas have been reported [13,16,18,22,23,24,25,27]. The results are encouraging, with tumor control rates >90% and a very limited toxicity to the anterior optic pathway. Namely, authors from different centers demonstrated that it is possible to treat meningiomas in proximity, or even encasing the optic nerve and chiasm, with mSRS. They showed a limited risk of radiation-induced optic neuropathy, which occurred in a <3% of the cases and only in patients with pre-treatment compromised function. Furthermore, after treatment, most patients showed a recovery of visual deficits. 

To the best of our knowledge, this is the largest series on the treatment of skull base meningiomas using multisession SRS. The technique appears to be effective, with progression-free survival >80% at 10 years. This is in line with the results of conventionally fractionated radiotherapy for the treatment of meningiomas. Multisession SRS also shows a very limited toxicity, represented mostly by transient trigeminal neuralgia (5.8%). Actually, no patient reported new visual deficits, with only two patients reporting severe edema (0.6%) and carotid occlusion (0.6%) attributed to tumor swelling. Furthermore, mSRS can provide the distinct advantage over FSRT of a limited number of sessions with greater comfort for patients, together with a lower overall integral dose to the normal brain tissues as a result of the radiosurgical definition of the PTV. 

### 4.3. Study Limitations

The main limit of this study lies in its retrospective nature, which could have affected the results, generating selection bias. Some aspects of the clinical outcomes, such as minor toxicities, may not have been adequately explored. In addition, patients with relapse might have consult different healthcare providers, leading to the loss of relevant long-term follow-up data. On the other hand, the strength of the study is represented by the unique records of relatively long radiological and ophthalmological follow-up data. In addition, this study was restricted to WHO Grade I meningiomas, because our strategy of treatment of atypical and anaplastic meningioma is different and requires higher doses and single fractions in most instances [52]. In 107/156 cases, this diagnosis could be only supposed on the base of precise radiological criteria, but it turned out to be wrong in two cases, which were excluded from the analysis. Thus, we cannot exclude, at this time, that other meningiomas in this series may show a later aggressive behavior. Finally, hypofractionation can theoretically select radioresistant [53] or even aggressive cell clones. Nevertheless, this remains a theoretical risk that is counterbalanced by the advantages offered by hypofractionation, consisting of lower exposure to and potential repair from radiation damage of normal tissue in complex skull base meningiomas. It should be also considered that mSRS represents an alternative to conventionally fractionated radiotherapy rather than to sSRS. 

## 5. Conclusions

Multisession stereotactic radiosurgery provides a safe and effective profile in the treatment of skull base meningiomas. Satisfactory local control and a low toxicity rate, together with patient comfort from a frameless procedure, suggest that mSRS should be considered the preferred radiotherapy treatment modality for large skull base meningiomas, or for meningiomas that grow close to the anterior optic pathway or other radiosensitive structures.

## Figures and Tables

**Table 1:** Patient clinical characteristics. ACF—anterior crania fossa; LSW—lateral sphenoid wing; PCF—posterior cranial fossa.

Variations	Value
**No. of Patients**	156
**Gender (M/F)**	37/119
**Age at the treatment time (years)**	
Mean (range)	58 ± 11.4 (30–81)
**Follow-up (months)**	
Mean (±SD) Median (range)	36.2 (±29.1) 36.6 (2–137)
**KPS**	
Mean (±SD) Median (range)	88 (±9.1) 90 (70–100)
**Treatment Modality**	
Upfront Adjuvant Residual Recurrence	107 49 22 27
**Tumor Site**	
Perioptic/ACF/LSW PetroClival PCF Cavernous sinus	72 (46.1%) 46 (28.5%) 15 (9.4%) 23 (14.7%)
**Pretreatment Cranial Nerves deficit**	35 (22.4%)
II III–IV–VI V VII VIII IX–X	13 (8.3%) 7 (4.5%) 9 (5.8%) 3 (1.9%) 2 (1.3%) 1 (0.6%)
**Histology**	
Yes	49 (31.4%)

**Table 2:** Treatments and dosimetric features.

Variations	Value
**Patients**	156
**PTV**	
Mean ± SD Median	10.3 ± 11.9 mL 7.5 mL
**Dose**	
Mean ± SD Median	25 ± 5.3 Gy 25 Gy
**Number of Fractions**	
Mean ± SD Median	5 ± 2 5
**BED (Gy)**	
Mean ± SD	87.2 ± 15.1
Median	87.5
**EqD2 (Gy)**	
Mean ± SD	43.6 ± 7.6
Median	43.8
**New Conformality Index**	
Mean ± SD	1.5 ± 0.3
Median (range)	1.4
**Prescription Isodose**	
Mean ± SD	76.5 ± 5.1
Median	77

## References

[1] DiBiase S.J., Kwok Y., Yovino S., Arena C., Naqvi S., Temple R., Regine W.F., Amin P., Guo C., Chin L.S. (2004). Factors predicting local tumor control after gamma knife stereotactic radiosurgery for benign intracranial meningiomas. Int. J. Radiat. Oncol. Biol. Phys..

[2] Hasegawa T., Kida Y., Yoshimoto M., Koike J., Iizuka H., Ishii D. (2007). Long-term outcomes of Gamma Knife surgery for cavernous sinus meningioma. J. Neurosurg..

[3] Iwai Y., Yamanaka K., Ikeda H. (2008). Gamma Knife radiosurgery for skull base meningioma: Long-term results of low-dose treatment. J. Neurosurg..

[4] Liscak R., Kollová A., Vladyka V., Šimonová G., Novotný J. (2004). Gamma Knife Radiosurgery of Skull Base Meningiomas. Acta Neurochir. Suppl..

[5] Kondziolka D., Mathieu D., Lunsford L.D., Martin J.J., Madhok R., Niranjan A., Flickinger J. (2008). Radiosurgery as definitive management of intracranial meningiomas. Neurosurgery.

[6] Kreil W., Luggin J., Fuchs I., Weigl V., Eustacchio S., Papaefthymiou G. (2005). Long term experience of gamma knife radiosurgery for benign skull base meningiomas. J. Neurol. Neurosurg. Psychiatry.

[7] Lee J.Y.K., Niranjan A., McInerney J., Kondziolka D., Flickinger J.C., Lunsford L.D. (2002). Stereotactic radiosurgery providing long-term tumor control of cavernous sinus meningiomas. J. Neurosurg..

[8] Minniti G., Amichetti M., Maurizi E.R. (2009). Radiotherapy and radiosurgery for benign skull base meningiomas. Radiat. Oncol..

[9] Nicolato A., Foroni R., Alessandrini F., Maluta S., Bricolo A., Gerosa M. (2002). The role of Gamma Knife radiosurgery in the management of cavernous sinus meningiomas. Int. J. Radiat. Oncol. Biol. Phys..

[10] Stafford S.L., Pollock B.E., Foote R.L., Link M.J., Gorman D.A., Schomberg P.J., Leavitt J.A. (2001). Meningioma radiosurgery: Tumor control, outcomes, and complications among 190 consecutive patients. Neurosurgery.

[11] Starke R.M., Przybylowski C.J., Sugoto M., Fezeu F., Awad A.J., Ding D., Nguyen J.H., Sheehan J.P. (2015). Gamma Knife radiosurgery of large skull base meningiomas. J. Neurosurg..

[12] Kondziolka D., Niranjan A., Lunsford L.D., Flickinger J.C. (1999). Stereotactic radiosurgery for meningiomas. Neurosurg. Clin. N. Am..

[13] Conti A., Pontoriero A., Midili F., Iatì G., Siragusa C., Tomasello C., La Torre D., Cardali S.M., Pergolizzi S., De Renzis C. (2015). CyberKnife multisession stereotactic radiosurgery and hypofractionated stereotactic radiotherapy for perioptic meningiomas: Intermediate-term results and radiobiological considerations. SpringerPlus.

[14] Conti A., Pontoriero A., Salamone I., Siragusa C., Midili F., La Torre D., Calisto A., Granata F., Romanelli P., De Renzis C. (2009). Protecting venous structures during radiosurgery for parasagittal meningiomas. Neurosurg. Focus.

[15] Conti A., Pontoriero A., Siddi F., Iatì G., Cardali S., Angileri F.F., Granata F., Pergolizzi S., Germanò A., Francesco T. (2016). Post-Treatment Edema after Meningioma Radiosurgery is a Predictable Complication. Cureus.

[16] Adler J.R., Gibbs I.C., Puataweepong P., Chang S.D. (2008). Visual field preservation after multisession cyberknife radiosurgery for perioptic lesions. Neurosurgery.

[17] Choi C.Y.H., Soltys S.G., Gibbs I.C., Harsh G.R., Jackson P., Lieberson R.E., Chang S.D., Adler J.R. (2010). Cyberknife Stereotactic Radiosurgery for Treatment of Atypical (Who Grade II) Cranial Meningiomas. Neurosurgery.

[18] Colombo F., Casentini L., Cavedon C., Scalchi P., Cora S., Francescon P. (2009). Cyberknife radiosurgery for benign meningiomas: Short-term results in 199 patients. Neurosurgery.

[19] Manabe Y., Murai T., Ogino H., Tamura T., Iwabuchi M., Mori Y., Iwata H., Suzuki H., Shibamoto Y. (2017). CyberKnife Stereotactic Radiosurgery and Hypofractionated Stereotactic Radiotherapy as First-line Treatments for Imaging-diagnosed Intracranial Meningiomas. Neurol. Med. Chir..

[20] Patil C.G., Hoang S., Borchers D.J., Sakamoto G., Soltys S.G., Gibbs I.C., Harsh G.R., Chang S.D., Adler J.R. (2008). Predictors of peritumoral edema after stereotactic radiosurgery of supratentorial meningiomas. Neurosurgery.

[21] Pham C.J., Chang S.D., Gibbs I.C., Jones P., Heilbrun M.P., Adler J.R. (2004). Preliminary visual field preservation after staged CyberKnife radiosurgery for perioptic lesions. Neurosurgery.

[22] Romanelli P., Bianchi L., Muacevic A., Beltramo G. (2011). Staged image guided robotic radiosurgery for optic nerve sheath meningiomas. Comput. Aided Surg..

[23] Romanelli P., Wowra B., Muacevic A. (2007). Multisession CyberKnife radiosurgery for optic nerve sheath meningiomas. Neurosurg. Focus.

[24] Marchetti M., Bianchi S., Milanesi I., Bergantin A., Bianchi L., Broggi G., Fariselli L. (2011). Multisession radiosurgery for optic nerve sheath meningiomas—An effective option: Preliminary results of a single-center experience. Neurosurgery.

[25] Marchetti M., Bianchi S., Pinzi V., Tramacere I., Fumagalli M.L., Milanesi I.M., Ferroli P., Frazini A., Saini M., DiMeco F. (2016). Multisession Radiosurgery for Sellar and Parasellar Benign Meningiomas: Long-term Tumor Growth Control and Visual Outcome. Neurosurgery.

[26] Shrieve D.C., Hazard L., Boucher K., Jensen R.L. (2004). Dose fractionation in stereotactic radiotherapy for parasellar meningiomas: Radiobiological considerations of efficacy and optic nerve tolerance. J. Neurosurg..

[27] Marchetti M., Conti A., Beltramo G., Pinzi V., Pontoriero A., Tramacere I., Senger C., Pergolizzi S., Fariselli L. (2019). Multisession radiosurgery for perioptic meningiomas: Medium-to-long term results from a CyberKnife cooperative study. J. Neuro Oncol..

[28] Paddick I. (2000). A simple scoring ratio to index the conformity of radiosurgical treatment plans. J. Neurosurg..

[29] Combs S.E., Adeberg S., Dittmar J.-O., Welzel T., Rieken S., Habermehl D., Huber P.E., Debus J. (2013). Skull base meningiomas: Long-term results and patient self-reported outcome in 507 patients treated with fractionated stereotactic radiotherapy (FSRT) or intensity modulated radiotherapy (IMRT). Radiother. Oncol..

[30] Kaul D., Budach V., Wurm R., Grün A., Graaf L., Habbel P., Badakhshi H. (2014). Linac-based stereotactic radiotherapy and radiosurgery in patients with meningioma. Radiat. Oncol..

[31] Solda F., Wharram B., Gunapala R., Brada M. (2012). Fractionated Stereotactic Conformal Radiotherapy for Optic Nerve Sheath Meningiomas. Clin. Oncol..

[32] Steinvorth S., Welzel G., Fuss M., Debus J., Wildermuth S., Wannenmacher M., Wenz F. (2003). Neuropsychological outcome after fractionated stereotactic radiotherapy (FSRT) for base of skull meningiomas: A prospective 1-year follow-up. Radiother. Oncol..

[33] Fokas E., Henzel M., Surber G., Hamm K., Engenhart-Cabillic R. (2014). Stereotactic Radiation Therapy for Benign Meningioma: Long-Term Outcome in 318 Patients. Int. J. Radiat. Oncol. Biol. Phys..

[34] Andrews D.W., Faroozan R., Yang B.P., Hudes R.S., Werner-Wasik M., Kim S.M., Sergott R.C., Savino P.J., Shields J., Shields C. (2002). Fractionated stereotactic radiotherapy for the treatment of optic nerve sheath meningiomas: Preliminary observations of 33 optic nerves in 30 patients with historical comparison to observation with or without prior surgery. Neurosurgery.

[35] Combs S.E., Ganswindt U., Foote R.L., Kondziolka D., Tonn J.-C. (2012). State-of-the-art treatment alternatives for base of skull meningiomas: Complementing and controversial indications for neurosurgery, stereotactic and robotic based radiosurgery or modern fractionated radiation techniques. Radiat. Oncol..

[36] Astner S.T., Theodorou M., Dobrei-Ciuchendea M., Auer F., Kopp C., Molls M., Grosu A.-L. (2010). Tumor Shrinkage Assessed by Volumetric MRI in the Long-Term Follow-Up after Stereotactic Radiotherapy of Meningiomas. Strahlenther. Onkol..

[37] Milker-Zabel S., Bois A.Z.-D., Henze M., Huber P., Schulz-Ertner D., Hoess A., Haberkorn U., Debus J. (2006). Improved target volume definition for fractionated stereotactic radiotherapy in patients with intracranial meningiomas by correlation of CT, MRI, and [68Ga]-DOTATOC-PET. Int. J. Radiat. Oncol. Biol. Phys..

[38] Jeremic B., Pitz S. (2007). Primary optic nerve sheath meningioma: Stereotactic fractionated radiation therapy as an emerging treatment of choice. Cancer.

[39] Pacelli R., Cella L., Conson M., Tranfa F., Strianese D., Liuzzi R., Solla R., Farella A., Salvatore M., Bonavolontà G. (2011). Fractionated Stereotactic Radiation Therapy for Orbital Optic Nerve Sheath Meningioma—A Single Institution Experience and a Short Review of the Literature. J. Radiat. Res..

[40] Afshar-Oromieh A., Wolf M.B., Kratochwil C., Giesel F.L., Combs S.E., Dimitrakopoulou-Strauss A., Gnirs R., Roethke M.C., Schlemmer H.P., Haberkorn U. (2015). Comparison of 68Ga-DOTATOC-PET/CT and PET/MRI hybrid systems in patients with cranial meningioma: Initial results. Neuro Oncol..

[41] Farzin M., Molls M., Kampfer S., Astner S.T., Schneider R., Roth K., Dobrei M., Combs S.E., Straube C. (2016). Optic toxicity in radiation treatment of meningioma: A retrospective study in 213 patients. J. Neuro Oncol..

[42] Mozes P., Dittmar J.-O., Habermehl D., Tonndorf-Martini E., Hideghéty K., Dittmar A., Debus J., Combs S.E. (2016). Volumetric response of intracranial meningioma after photon or particle irradiation. Acta Oncol..

[43] Stade F., Dittmar J.-O., Jäkel O., Kratochwil C., Haberkorn U., Debus J., Combs S.E. (2018). Influence of 68Ga-DOTATOC on sparing of normal tissue for radiation therapy of skull base meningioma: Differential impact of photon and proton radiotherapy. Radiat. Oncol..

[44] Combs S.E., Farzin M., Boehmer J., Oehlke O., Molls M., Debus J., Grosu A.-L. (2018). Clinical outcome after high-precision radiotherapy for skull base meningiomas: Pooled data from three large German centers for radiation oncology. Radiother. Oncol..

[45] Kessel K.A., Fischer H., Oechnser M., Zimmer C., Meyer B., Combs S.E. (2017). High-precision radiotherapy for meningiomas: Long-term results and patient-reported outcome (PRO). Strahlenther Onkol..

[46] Lo S.S., Cho K.H., Hall W.A., Kossow R.J., Hernandez W.L., McCollow K.K., Gerbi B.J., Higgins P.D., Lee C.K., Dusenbery K.E. (2002). Single dose versus fractionated stereotactic radiotherapy for meningiomas. Can. J. Neurol. Sci..

[47] Metellus P., Regis J., Muracciole X., Fuentes S., Dufour H., Nanni I., Chinot O., Martin P.-M., Grisoli F. (2005). Evaluation of Fractionated Radiotherapy and Gamma Knife Radiosurgery in Cavernous Sinus Meningiomas: Treatment Strategy. Neurosurgery.

[48] Torres R.C., Frighetto L., De Salles A.A.F., Goss B., Medin P., Solberg T., Ford J.M., Selch M. (2003). Radiosurgery and stereotactic radiotherapy for intracranial meningiomas. Neurosurg. Focus.

[49] Conti A., Romanelli P., Pantelis E., Soltys S.G., Cho Y.H., Lim M. (2020). CyberKnife NeuroRadiosurgery A Practical Guide.

[50] Girvigian M.R., Chen J.C., Rahimian J., Miller M.J., Tome M. (2008). Comparison of early complications for patients with convexity and parasagittal meningiomas treated with either stereotactic radiosurgery or fractionated stereotactic radiotherapy. Neurosurgery.

[51] Unger K.R., Lominska C.E., Chanyasulkit J., Randolph-Jackson P., White R.L., Aulisi E., Jacobson J., Jean W.C., Gagnon G.J. (2011). Risk Factors for Posttreatment Edema in Patients Treated With Stereotactic Radiosurgery for Meningiomas. Neurosurgery.

[52] Acker G., Meinert F., Conti A., Kufeld M., Jelgersma C., Nguyen P., Kluge A., Lukas M., Loebel F., Pasemann D. (2019). Image-Guided Robotic Radiosurgery for Treatment of Recurrent Grade II and III Meningiomas. A Single-Center Study. World Neurosurg..

[53] Nahum A.E. (2015). The Radiobiology of Hypofractionation. Clin. Oncol..

